# Local Sport Event Policies and Sustainability: A Puzzle Approach

**DOI:** 10.3389/fspor.2021.667762

**Published:** 2021-12-16

**Authors:** Christian Tolstrup Jensen

**Affiliations:** ^1^University of South-Eastern Norway, Kongsberg, Norway; ^2^Malmö University, Malmö, Sweden

**Keywords:** events, sport, policy, local, municipalities, sustainability, Norway, SDGs

## Abstract

As demands for more sustainable ways of living increase, organisers of sport events have come under increasing pressure to adapt. At the same time, more and more national and local event policies increase the demand for events. These two trends raise the question of how policy makers can combine the demand for events with a sustainable way of living; a question that so far has been subject to little research. The present paper analyses the conceptualisation of sustainability in all local policies relating to events in Norwegian municipalities. The paper is based on the analysis of policies covering 22 municipalities and includes both general development plans and more specific policies on events in its analysis. The analysis shows that all the municipalities have adopted a “broad” conceptualisation of sustainability, i.e., pursued a development, which should not limit the possibilities of future generations, in their general development plans. Although the general development plans serve as a basis for every other policy, the paper also shows that the municipalities in the specific policies for events often had “narrow” conceptualisation of sustainability, i.e., focusing on making local events reoccurring and/or increasing the capacity for hosting external events. The findings emphasise the relevance of looking at the local level when conducting future studies on events and sustainability and suggest that the practitioners acknowledge the complexity of reconciling demands for more events and increased sustainability.

## Introduction

In today's society, organisers of sport events and other large social gatherings are under pressure to find new sustainable ways of staging their events just like the rest of society. On the global level, owners of the biggest sport events like the International Olympic Committee (IOC) and the international football federation, FIFA, have launched initiatives for reducing their environmental impact (Mallen et al., 2011, pp. 241–242; cf. IOC, 2014). Even non-sport organisations such as the United Nations (UN) have promoted new international frameworks for sport's sustainability (ISO, 2012; Buscarini et al., 2021; ISO/TC 292).

Such frameworks are all the more relevant as there is little research suggesting that events currently contribute to sustainable societies when compared to the research suggesting the opposite especially with regard to sport mega events ( Lenskyj, 2004; Preuss, 2007; O'Brien and Chalip, 2008; cf. e.g., Collins et al., 2009; Smith, 2009; Mallen et al., 2011; Mair and Whitford, 2013; Zimbalist, 2015; Baade and Matheson, 2016; Zimbalist, 2017; Koenigstorfer et al., 2019; Thomson et al., 2019). Considering research on smaller events, it has been discussed whether these events could be more sustainable (Taks, 2013, 2016). However, other studies show that these events can also have negative consequences and see a need for more research on best practises and policies (Higham, 2018, p. 68; Lindsey and Darby, 2019; Jiménez-García et al., 2020; Melo et al., 2021, p. 37).

The research interest in small events has also led to a focus on events in series. It has, first and foremost, been concerned with the study of event portfolios, but there have also appeared studies on the spread of event hosting strategies often focusing on smaller events (Ziakas, 2010; Stopper et al., 2011b; Chappelet and Lee, 2016; Andersson et al., 2017; Antchak, 2017; Antchak et al., 2019). Such strategies could be seen as concerned with sustainability in two ways. At their core, the strategies are meant to allow for a continuous run of events and a stable outcome for the local host community (Gibson et al., 2012; Taks, 2013; Clark and Misener, 2015; Antchak, 2017; Kim, 2020, Chapter 3). However, (and potentially in conflict with the first), given the general demand for building sustainable societies, they should ideally also be compatible with the holistic idea of sustainability promoted by, for instance, the UN in their Sustainable Development Goals (SDGs). Right now, research on sport event policies has yet to look into how the policies handle these two conceptualisations of sustainability, which will be developed further in the next section (McCloy, 2009; Leopkey et al., 2010; Stopper et al., 2011a,b; Chappelet and Lee, 2016; Pinson, 2016; Schnitzer et al., 2017; Leopkey and Ellis, 2019). Vassilios Ziakas, who coined the term “event portfolio,” for instance, only recently encouraged researchers and practitioners to adopt a “holistic” approach emphasising sustainability as “the triple-bottom-line of economic, social and environmental prosperity” when analysing or developing event portfolios (Ziakas, 2019, p. 29). Comparing the state of the art in sport event strategy research with the research on singular events, one could look to urban geographer Andrew Smith, who, in 2012, discussed how singular events could give leverage to other policies in a host community (Smith, 2012, p. 14; cf. Chalip, 2014). In 2021, research on event policies might be in similar position raising the question: How do or should event strategies work in tandem with other policies?

Answering this question would move the research field forward and has a significant practical relevance compared to studies of single events. After all, improving the sustainability of the event strategies or event policies has the potential to regulate the impact of not just one but several events.

As a contribution to our understanding of sustainability in sport event hosting strategies, the aim of the present paper is to give insight into how (sport) event policies currently conceptualise sustainability in a specific (Norwegian) context and discuss the potential reasons for and the implications of such conceptualisation(s) for practitioners and future research^1^.

The first part of the paper introduces the foundation for the paper's analysis and discussion. It begins by outlining the two conceptualisations of sustainability that serves as a basis for the analysis. Afterwards, the theoretical frame for the paper's discussion is introduced making the case that policy development functions as a “puzzle.” This theory is closely related with the final part of the introduction that presents the context for the policies analysed in the paper. The second part presents the results of the study before the paper concludes with a discussion.

### Conceptualising Sustainability: A Broad and a Narrow Concept

As hinted in the paper's introduction, the increased interest in sustainability affects most parts of society. In a recent paper, McCullough et al. (2020) suggests that “sustainable” has become “perhaps the most important buzzword in contemporary global policy” (McCullough et al., 2020, p. 510). The introduction, however, also suggests that the meaning of this buzzword vary. Extending on this assumption, this section argues that a conceptualisation of “sustainability,” depending on the context, is either broad or narrow.

The paper's broad conceptualisation is derived from the World Commission on Environment and Development's definition of sustainable development as a “development that meets the needs of the present without compromising the ability of future generations to meet their own needs” (World Commission on Environment Development, 1987, Chapter 2.I.1). Although the definition has been criticised for being vague (Borowy, 2018, p. 155), it is commonly referred to in literature on sustainability and sport events as well as in general discussions on sustainable development (e.g., cf. Bell and Morse, 2018, p. 188; Caradonna, 2018; McCullough and Kellison, 2018; Escher, 2020; Kim, 2020; Triantafyllidis and Darvin, 2021). Perhaps because of its vagueness, the definition from the commission has been followed up by several more specific and operational framework for evaluating the sustainability of a certain action or society (Bell and Morse, 2018; cf. Kim, 2020, Sec. 2.2.4). Currently, the most prominent of these frameworks is probably the UN's SDGs United Nations. The point of the SDGs is to become common points of references to every nation and potentially for event policies too. Indeed, some goals like Goal 11 (“Sustainable cities”) and Goal 12 (“Responsible consumption and production”) have already been used as a frame for analysing the local impacts of large sport events (Buscarini et al., 2021; Triantafyllidis and Darvin, 2021).

The triple bottom line is another older but still prevalent sustainability framework especially for businesses and organisations that assesses the sustainability of, e.g., an organisation according to its economic, environmental and social impacts (Elkington, 2004; Purvis et al., 2019).

Finally, in 2017, the economist Kate Raworth suggested a third model for how to conceptualise or imagine the borders within which sustainable development could take place. Her idea, the “doughnut economy,” is that sustainable development means to stay within the limits of the doughnut not producing too little to sustain everyone's livelihood nor overproducing and thereby exceeding Earth's ecological limited (Raworth, 2017).

All these concepts for achieving sustainable development have a holistic view in common as they target society as a whole (the SDGs) or encourage individual organisations to consider the society of which they are part (the triple bottom line). In this paper, the idea that sustainability requires a holistic view of society and a broad conceptualisation of sustainability is called on.

These models for a broad conceptualisation of sustainability are of relatively recent origin considering that, “sustainability” (or “berekraft” in Norwegian) did not only enter the Norwegian language with the report from the World Commission on Environment and Development. Rather, the report and the discussions it inspired only added a new meaning to at least two existing understandings of “berekraft.” According to one of these understandings, “berekraft” is an adjective describing a literal ability to support something physically, like a foundation's ability to support a building. Finally, “berekraft” can also be used to describe something's ability to last figuratively (Det Norske Akademis ordbok; Språkrådet and Universitetet i Bergen). Studies on the use of the English term “sustainability” shows that a similar meaning exists in English. All in all, “[a]s a historical endeavour, sustainability concerns the long-term success of problem-solving efforts,” leaving us with the question: what is the problem “sustainability” is meant to solve in a given context (Tainter, 2018, p. 40)?

The first original understanding of berekraft/sustainability is for instance concerned with the problem of whether a bridge is able to handle heavy loads. The problem in the second meaning is about securing a lasting outcome (Dale, 2018, p. 76). In the last century, this outcome has often equalled steady economic growth given the prominent idea of economic growth as a “universal remedy for some of the most pressing challenges of modern societies” (Schmelzer, 2018, p. 171). This, however, is an understanding that could be seen as conflicting with the broad conceptualisation of sustainable development (cf. Higham, 2018, Chapter 4). This means that the conceptualisation of sustainability as a solution to the specific problem of establishing a stable (economic) outcome stands in contrast to the broad conceptualisation and represents consequently the paper's narrow conceptualisation of sustainability. In the present paper, a narrow characteristic would, for instance, apply to a policy aiming specifically at making events in a municipality a common occurrence and, ideally, also self-supported. It is also a conceptualisation which is reflected in Chappelet and Lee (2016)'s study on sport event policies when suggesting that sport event hosting strategies are “employed for the successful bidding and hosting of sport event*s*” (note the plural) (Chappelet and Lee, 2016, p. 36). As a frame for discussing why a certain conceptualisation might come about, the following section introduces the paper's theoretical framework.

## Theory

To enable a discussion of the presence of a given conceptualisation of sustainability as dependent on the problem the conceptualisation is meant to solve, the paper's theoretical point of departure is that an organisation develops policies to solve problems (Dunn, 2018, p. 5). However, this does not mean that there is a direct line between the solution (the policy) and the problem at hand. Instead, the paper considers event policies to be outcomes of processes influenced by other ideas, agendas, policies, etc. In other words, policy development is complex—a view that existing research on policies related to sport events as well as sport and sustainability supports (Leopkey et al., 2010, p. 128; Chappelet and Lee, 2016, p. 5; Lindsey and Darby, 2019).

The complexity arises not only due to the very number of stakeholders but also due to the potential disagreements on what the problem is. While there might be general agreement on for instance the need to have a tourism policy, some might want to regulate tourism flows (problem: there are too many tourists), whereas others want to attract more tourists (problem: there are too few tourists). Explaining that the aim of a given policy therefore only not requires access to the policy but also “knowledge about the antecedent conditions” (Dunn, 2018, p. 5), which will be discussed in the next section.

Before that, looking more specifically at how to analyse and discuss the role of sustainability in the sport event policies, the paper draws on the ideas of sociologist, Christopher Winship, who argues that traditional policy evaluations aiming at finding the best solutions to a specific problem have difficulties in cases “with multiple and conflicting ends” (Winship, 2006, p. 110). Winship here is inspired by, among other things, the idea of “wicked problems,” that is, problems that are inter alia characterised by their lack of definitive formulations since “the formulation of a wicked problem is the problem” and also, eventually, definitive solutions (Rittel and Webber, 1973, p. 161). How, for instance, can one formulate a simple problem whose solution would improve peoples' life definitively (Rittel and Webber, 1973, p. 167)? Formulating this problem in simple soluble terms is very difficult, if not impossible, and instead of finding a definitive solution, one should look for ways to improve the conditions or mitigate the problem (and thereby potentially causing new problems) (Rittel and Webber, 1973, pp. 162–163). Based on this complexity, Winship suggests that politicians and researchers see policy developments as puzzles. By assembling the puzzle in new ways or, allegedly, breaking the idea of the typical puzzle, and adding, revising, or removing pieces from the puzzle, it is possible to figure “out how to rectify a set of seemingly conflicting policy ends” (Winship, 2006, p. 119). Even if the stakeholders do not know what the solved puzzle looks like in the end, they know when it appears coherent.

In the present case, I suppose sport event policies can have at least two (potentially) conflicting policy ends. Based on the puzzle theory, the paper concludes with a discussion on how local event policies currently seem to overcome this gap between “non-commensurable world-views” (Winship, 2006, p. 116). As already mentioned, this discussion, however, needs to be informed by the policies' “antecedent conditions,” which the next section introduces.

## The Context For Sport Events Policies in Norway

This section contextualises the analysed policies with a focus on the role of the public administration and the organised sport in Norway as the primary stakeholders in Norwegian sport events (Jensen, 2020).

The paper's focus on local event policies makes the municipalities a natural starting point for the overview. However, the municipalities are also a relevant starting point since the public administration in Norway is generally decentralised giving the municipalities much of the responsibility for Norway's sustainable development (Amundsen et al., 2018; Reed, 2018; Wang et al., 2018; Hansen, 2020). Still, the municipalities also negotiate with other municipalities and public administrations on the regional and the national levels (Hanssen). In 2019, for instance, the Norwegian government required that all Norwegian municipalities make the SDGs a guiding principle in their master plans (Kommunal- og moderniseringsdepartementet, 2019). By then, the local awareness of the SDGs would already have been quite high. In 2018, 84% of 51 municipalities participating in a survey found that the SDGs were important, and one in four had plans for how to implement them (Deloitte, 2018, p. 11). Perhaps less surprisingly, a survey of 22 municipality master plans in 2020 showed that 14 of them already had implemented the SDGs (Seim in Singsaas, 2020). Despite the small samples (in 2018 Norway had 430 municipalities, in 2020 there were 354), the findings indicate a local support for the SDGs even prior to the adoption of the national policy paper. A support, which also would indicate that the global debate is also influential in the local public administration. However, these various policies and initiatives also are subject to local hearings and at least one study focusing on the transportation and area policies in the Oslo has shown that sustainability in that case was a “wicked problem” (Fossheim and Nazareno, 2020). We will come back to how local hearings also influenced the final shape of the local event policies in the result section.

Since Norway does not have a national event policy, the national influence on sport events hosting seems limited compared to the influence found in some other countries (Chappelet and Lee, 2016, p. 11). Instead of a central policy, the national influence is scattered across several policies and regulations. The white paper on tourism is one such thing which notes that sustainability is an overall aim and encourages event tourism (Nærings- og Fiskeridepartementet, 2017). An aim, which national sport policy, however, does not repeat in its section on events (Kulturdepartementet, 2012, pp. 114–115).

Despite the lack of a national event policy, there has, however, been an unofficial practise of providing national financial support for international sport events (Lechner and Solberg, 2021), which the government formalised to a certain degree in 2019 by launching a “test”-event policy. Among other things, the policy asks the host organisations to “consider sustainability” when applying for financial support without providing additional details (Kulturdepartementet, 2019). Furthermore, Innovation Norway, a publicly owned business advisory company, proposed a national event policy in December 2019, which also emphasises the need for events to be sustainable (Innovasjon Norge, 2019).

In short, policies regulating events in Norway are mainly the municipalities' domain—presumably considering inputs from public partners as well as private stakeholders. Relevant private stakeholders, when speaking of sport events, could, for instance, be national governing bodies (NGBs) and local sport associations. Their inputs related to sustainability in a broad sense could however very well be negligible since a report in 2018 described the Norwegian NGBs' work on sustainability—on a national average—as “moderate” (Geeraert, 2018, p. 172; cf. Goldblatt, 2020, p. 16). Considering the five biggest sport federations' general strategy documents in greater detail confirms this conclusion as only the football federation has sustainability as an overall aim; although the skiing federation discusses the impact of the climate changes on future competitions (Norges Skiforbund, 2016, p. 20; Norges Gym- og Turnforbund, 2018; Norges Handballforbund, 2019, p. 6; Norges Fotballforbund, 2020, p. 4; cf. Norges Golfforbund, 2020). The national confederation of sport in Norway (Norges Idrettsforbund, NIF), however, has sustainability in a broad sense as part of its aims (NIF, 2019).

Still, the NGBs might influence the importance give to sustainability conceptualised narrowly related to sport events, as they all—except the Golf federation—discuss the role events play for their development. The gymnastics, for instance, is seeking to improve the “event quality” of their national senior championship (p. 15) and the skiing federation wants to sustain “Norway's leading position as leading nation and organiser” by hosting international events in all its disciplines (Norges Skiforbund, 2016, p. 25; Norges Gym- og Turnforbund, 2018, p. 12).

**Figure 1:**
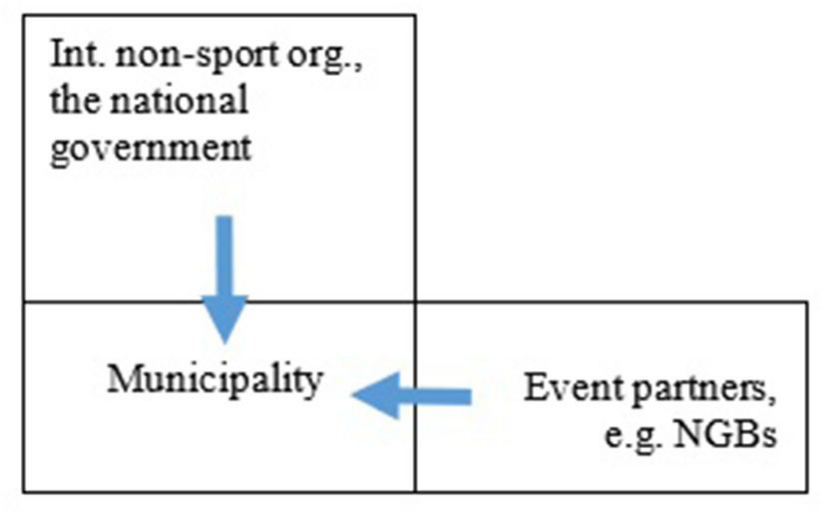
Outline of the theoretical position of a municipality developing an event policy with inputs from above and from their event partners, e.g., the sport organisations.

Summing up, the contextual section has introduced two main sources of influence to consider in the analysis of the conceptualisations of sustainability. The first can be described as a horizontal relation between a host municipality and its event partners. The second source runs along a vertical axis between the municipality, the national government, and international non-sport organisations (cf. Figure 1). In the discussion, we will come back to the question whether this initial model can account for the paper's results. Therefore, it is important to note that a municipality negotiate with their partners and seek to influence this network. The one-way arrows in Figure 1 are therefore too simple for a diachronic analysis. The aim of this study, however, is only to determine the influence on the municipalities and their concept of sustainability reflected in their *current* policies.

## Method

The theory outlined policy development as a complex phenomenon. The contextual section supported this hypothesis and with regard to the choice of method, complexity invites for a case study (Yin, 2018). As the aim of the paper is to give insight into how municipalities conceptualise sustainability in their event policies, the paper's case study however consists mainly of a document analysis.

### Material

The empirical data for the document analysis includes all event policies or proposals for such made public by any Norwegian municipality as of December 2020. This data was collected in two rounds. The first round consisted of a search on google.com for “arrangementsstrategi^2^” limited to all subdomains of kommune.no, the common domain for all Norwegian municipalities. The exact search string was “^*^.kommune.no + arrangementsstrategi” (without apostrophes).

In the second round, this crude data collection was supplemented with a manual search for event policies on each of homepages of the municipalities, for which the first search had returned no results but where it nevertheless seemed likely that the municipality would have an event policy given its size or earlier expressions of interest. This search, in particular, focused on the six municipalities,^3^ which participated in the development of the first Norwegian white paper on event tourism (Innovasjon Norge, 2011) and the ten biggest cities in Norway.

In total, the data collection yielded 15 results suitable for further analysis ranging from specific and implemented event policies to the mentioning of events as part of the municipality's master plan (cf. Table 1)^4^. The 15 results cover 13 municipalities and two regions (the Haugesund region^5^ and the Lillehammer region^6^) covering nine municipalities. The paper, therefore, analyses event polices relevant for 22 municipalities. Given that there are 356 municipalities in Norway, the study could sound limited, which is not the case considering the number of people affected by the policies. In total, these 22 municipalities amount to around 1.9 million inhabitants (~35 % of the Norwegian population).

**Table 1:** The data collected for the analysis of each of the municipalities.

**Municipality/region**	**Master plan analysed**	**Stand-alone event policy?^**a**^**	**State of event policy**	**Event policy effective since**	**Proceeding analysed**
Bergen	Yes	Yes	Approved policy	June, 2018	Yes
Drammen	Yes	No (Master plan)	Approved policy	2013	–
Fredrikstad	Yes	Yes	Approved policy	December, 2020	Yes
Halden	Yes	Yes	Approved policy	?	NA^b^
The Haugesund region	Yes	No (Business policy)	Suggested policy	June, 2020	–
Kristiansand	Yes	No (Master plan)	Suggested policy	May, 2020	–
The Lillehammer region	Yes	Yes	Approved policy	August, 2019	Yes
Oslo	Yes	Yes	Approved policy	May, 2019	Yes
Sandnes	Yes	Yes	Approved policy	2016^c^	Yes
Skien	Yes	No (Master plan)	Suggested policy	2013	–
Stavanger	Yes	Yes	Approved policy	2013	Yes
Sunnfjord	Yes	No (Business policy)	Approved policy	2019	–
Tromsø	Yes	Yes	Approved policy	2017	Yes
Trondheim	Yes	No (Master plan)	Approved policy	2009	–
Ulstein	Yes	No (Business policy)	Memo	2018	–

*^a^Does municipality have a stand-alone event policy or is the policy implemented within a broader plan for the whole of the municipality or region? ^b^The archival search yielded no results for Halden.*

*^c^The policy is stated to run 2016–2019, however, as of December 2020, the policy would still be presented as the official policy on the homepage of the Sandnes Municipality (Sandnes Kommune, 2020)*.

Table 1 lists the data considered in the analysis of each municipality with some additional information regarding the event policies. One sees for instance that several of the event policies are quite new. Event policies in Norway seem to enjoy political attention in line with the trend noted in the paper's introduction. The recent changes could however also have to do with a reform of the administrative regions in Norway taking effect on 1 January 2020.

As Table 1 shows, the analysis, in addition to the event policies, also considers the master plans for all the municipalities [regardless of whether the municipality's policy on events is covered in the master plan, a business policy or a specific event policy (cf. the sixth column)]^7^ and the proceedings prior to the adoption on the event policies. The aim is to gain a better insight into the influences on the local event policy in line with the policy theory. The data regarding the proceedings is however limited and the focus in the analysis will be on the event policies.

### Method of Analysis

The analysis of the policies is based on Glenn A. Bowen's partition of a document analysis into “skimming (superficial examination), reading (thorough examination), and interpretation” (Bowen, 2009, p. 32). The first step (covering skimming and reading) consists of reviewing the policy documents and marking relevant passages, i.e., those relevant for determining the means and ends of each policy and any mentioning of sustainability or event. The second step, “interpretation,” covers the analysis of the findings with the aim of characterising the typical ways of conceptualising sustainability in the policies.

While this method should yield relevant results, additional sources of data could have been useful for confirming the obtained information and explore the complexity of the case further [Yin (2018) cf. data triangulation (Johnson et al., 2007, pp. 114–115)]. However, interviews with municipality representatives could have come at the cost of the overview offered by the present overarching document analysis and would be better suited for particular studies. Targeted studies based on interviews and observations could, for instance, show whether local practises diverge significantly from the behaviour prescribed in the policies. In such a case, the current paper's findings would open for a discussion on pitfalls when implementing sustainability in event policies. The analysis, therefore, only considers the data to the Norwegian event policies and municipality master plans as described in the previous section.

## Results

The result section comes in three parts. In the first part, the conceptualisation of sustainability in the master plans is analysed. The focus here is to see if there are any general conceptualisations of sustainability, which the more targeted policies would have to consider. However, a more specific analysis of the conceptualisation of sustainability was not made in the three cases in which the municipalities express their interest in events in their master plans only.

In the last two parts of the results section, the results on the conceptualisation of sustainability related to events in the municipalities' business and event policies, respectively, is presented.

### Sustainability and Events in Local Master Plans

The analysis of the master plans shows that the 22 reviewed master plan all considered sustainability; the majority however only do so in a very vague sense.

Thus, only six of the master plans make an explicit link between sustainability and one of the common frameworks for sustainability conceptualised in a broad sense (cf. Table 2). Oslo and Frederikstad, for instance, make explicit references to the SDGs (Frederikstad Kommune, 2018, p. 8; Oslo Kommune, 2019, p. 3 cf. p. 11). Oslo also, as the only of the reviewed municipalities, makes a reference to the national policy paper, which asks the municipalities to incorporate the SDGs into their master plans (Kommunal- og moderniseringsdepartementet, 2019; Oslo Kommune, 2019, p. 11; cf.). This indicates the national level as a source of influence in addition to the global standards such as the SDGs and the triple bottom line, which the Kristiansand and Skien municipalities use to substantiate their conceptualisation of sustainability. Kristiansand for instance declares that the triple bottom line and the SDGs should be a foundation for every future decision. These aims are both “how they [: the municipality] want it” and a contribution to the global development (Skien Kommune, 2015; Kristiansand Kommune, 2020, p. 9; cf.). However, Kristiansand's overall policy, similar to the other five master plans, does not specify how these aims will affect the specific policy fields such as events, which the master plan mentions as an important area for further local development (Kristiansand Kommune, 2020, p. 7).

**Table 2:** The stand on sustainability in the master plans in every municipality included in the study.

***n* = 22^**a**^**	**Validity for each master plan**	**“SDG”**	**Triple bottom line**	**Otherwise including sustainability**
Bergen	2015–2030			Yes (promises to include “sustainability” in a wide range of its policies, p. 3)
Drammen	2013–2036			Yes (the vision being: “sustainable city growth” (p. 15)
Frederikstad	2018–2032	Yes		
Halden	2018–2050			Yes (it is possible to read its plan as compliant with a triple bottom line approach, p. 5)
The Haugesundregion	2015–2027 (Tysvær); 2014–2023 (Karmøy); 2014–2030 (Haugesund); 2017–2029 (Vindafjord); 2016–2026 (Etne); 2011–2023 (Sveio)			All find sustainability relevant for the planning. Some pay special attention to the economic or the environment implications (Tysvær, p. 36.38- Sveio, p. 11, 21). Others have a broad, generation-definition used in the Brundtland report (Etne p. 4) or make the triple bottom line explicit (Haugesund, p. 4).
Kristiansand	2020–2030	Yes		
The Lillehammerregion13F	2014–2025 [Øyer), 2014–2027 (Llh.), 2014–2026 (Gausdal)			The master plans discuss sustainability in relation to specific themes [Øyer: climate (p. 17), Gausdal: public health (p. 6) and Lillehammer: Norwegian law (p.4)^b^].
Oslo	2019–2040	Yes		
Sandnes	2019–2035			Yes (it is possible to read its plan as compliant with a triple bottom line approach, p. 5)
Skien	2015–2022		Yes	
Stavanger	2020–2034	Yes		
Sunnfjord	(founded 1/1 2020. No plan at the time of writing)			Sunnfjord however considered sustainability in some specific plans for e.g., the environment (Sunnfjord kommune, 2019).
Tromsø	2020–2032	Yes		
Trondheim	2009–2020			Yes (it is possible to read its plan as compliant with a triple bottom line approach, p. 14)
Ulstein	2017–2029			Yes (reference to the UN initiated Agenda 21)

*^a^The Haugesund and Lillehammer regions have regional event policies but no regional master plans. I have instead reviewed the master plans of the individual member municipalities (listed in the second column). Therefore n = 22 and not 15 (number of entities in column 1).*

*^b^At the time of the data collection the Lillehammer master plan however was under revision to accommodate a bigger emphasis on the SDG (Lillehammer Kommunestyre, 2020, no. 1/20)*.

The remaining nine master plans all mention “sustainability” too but do so without referring to a framework conceptualising sustainability broadly or—in a few cases—only with implicit references. Bergen is an example of the first, insofar, as it wants the city to have “a sustainable growth which considers the climate and the environment” (Bergen Kommune, 2015, p. 11). The plan, however, does not define what “growth” or “sustainability” mean beyond taking the climate and the environment into consideration. The plan from Halden exemplifies the latter as it sees “sustainability” as a response to climate change (environmental bottom line)—a factor when planning the future business logistics and “social sustainability” based on “bridging and bonding” as an aim for the development of the local community (Halden Kommune, 2018, pp. 5, 8, 22). The reference is implicit but for the initiated reader, it is clear that the Halden Municipality is referencing the triple bottom line approach. Similarly, the politicians in Trondheim want a “sustainable city,” which “plans for economic, social, and cultural growth considering the needs of today without destroying nature's future ecological sustainability” (Trondheim Kommune, 2009, p. 14).

Trondheim is also one of only three municipalities, which only expresses an interest in events in their master plans (the other two being Kristiansand and Skien, cf. Table 1). These expressions of interest are generally brief and general. In Trondheim's reference to events, it is possible to read an effort to make events socially sustainable into their event aims, cf., the aim of using events for “bringing about development, inclusion, participation and enthusiasm for Trondheim” (Trondheim Kommune, 2009, p. 25). In the cases of Skien and Kristiansand, events are simply important and none of the three cases have any details on how to handle events in a sustainable way regardless of how one conceptualises sustainability (Skien Kommune, 2015, p. 12; Kristiansand Kommune, 2020, p. 7).

On the one hand, the review of the masterplans shows that there is a widely recognised demand for policies based on sustainability in all the municipalities. A demand, which could affect other policies given that a master plan—as, for instance, in Bergen—sets the “long-term aims and strategies for the whole community in the municipality” (Bergen Kommune, 2015, p. 6). On the other hand, although the municipalities generally envision a broad conceptualisation of sustainability, only few municipalities indicate how they plan to implement their sustainability concept.

With regard to the further analysis, the aims of the master plans do not seem to be precise enough to determine the conceptualisations in the specific business or event policies analysed below. Considering only the master plans, the municipalities seem to dodge the dilemma between events and sustainability instead of reconciling the conflicting ends as suggested by the puzzle-theory.

### Sustainability and Events in Business Policies

Three of the municipalities (Haugesund, Ulstein and Sunnfjord) include their event policy in a business policy (cf., Table 1), and on an overall level, these policies restate the general focus on sustainability with connotations of a broad conceptualisation, which also appeared in the respective master plans. The plan for the Haugesund region, for example, conceptualises sustainability by paraphrasing the definition given in the Brundtland-report; echoing the definition used in the regional master plan (cf. Haugaland Vekst, 2017, p. 7; Haugaland Vekst, 2019, p. 18).

However, with regard to the relation between sport events and sustainability, the business policies do little to make the conceptualisation of sustainability from the master plan more specific. This might be because all the business plans see sport-based events as so important that the events need their own policies rendering any considerations temporary and thus of little use. Ulstein, for instance, hopes to develop a specific policy since it could improve the “coordination” of the events, Haugesund wants to increase the return on the public financial support, and Sunnfjord plans for a specific policy in order to increase its “professionalism” (Ulstein Kommune, 2018, p. 13; Sunnfjord Utvikling, 2019, p. 19; Karmøy Kommune, 2020, p. 5). Thus, in the future sport event policies, there seems to be support for a narrow conceptualisation, however, at least Ulstein imagines that (when eventually adopting an event policy) the improved coordination of the events will lead to a consideration of the triple bottom line (Ulstein Kommune, 2018, p. 6).

Finally, the analysis of the business policies also supports the initial claim that the interest in events is increasing rapidly. The regulation of events is merely stopping over on its way to an event policy. The risk of such rapid development is, of course, that the focus on making an efficient event hosting policy overtake the need for including a broad conceptualisation of sustainability in the policies. The final part of the results on the event policies will show whether this risk is real.

### Sustainability and Events in Event Policies

The data set contains eight local or regional event policies that are either approved or pending approval. In five of the cases (Bergen, Frederikstad, Sandnes, Stavanger and Tromsø), the conceptualisation of sustainability in the policies is related to the narrow conceptualisation. Here, the event policies should mainly help attracting more events to the municipalities. In other words, the policy makers had registered a deficiency of events in the municipalities and sought to resolve it by adopting an event policy. The policy for the Sandnes Municipality is the most clear example of this as it only mentions “sustainability” in the section on economy when explaining how well-established events “remain attractive because of their sustainability and continuity” (Sandnes Kommune, 2016, p. 7). Other policies propose founding forums for exchanging ideas or professionalising the municipality by lessening the amount of bureaucracy, making it easier for organisers to get access to the local facilities, etc. In the case of the Tromsø municipality, the event policy should even—in the end—make more events run “unsupported” by the public and yield a surplus (Tromsø Kommune, 2017, p. 3).

This is not to say the event policies focused only on the monetary aspect of the events. The policy for Tromsø also mentions the potential for events to increase the happiness of the participants and spectators (Tromsø Kommune, 2017, p. 4). This could be seen as a form of social sustainability. However, there is no mentioning of the third bottom line, the environmental dimension. Frederikstad similarly wants, while making the events professional, that events “should be staged in a safe way, which considers the local cultural heritage, nature, noise and safety” (Frederikstad Kommune, 2019, p. 7, cf. p. 16). Finally, Bergen's event policy refers to a general commitment in the business policy to the SDGs and introduces a procedure for certifying the environmental aims of each event (Bergen Kommune, 2018, pp. 5, 14). Still, the policy does not add any explicit demands for how the events should fulfil these aims. Events in Bergen should simply “create an active, attractive and unique event city that supports local value, growth, development, cooperation and experiences” (Bergen Kommune, 2018, p. 6).

Within the five cases without explicit references to sustainability in a broad sense, Tromsø and Bergen thus stand out as ambiguous as they have a potential for a broader conceptualisation of sustainability. Whether this is the case in praxis depends on further studies of the local event practises.

Conversely, the policies in Oslo, Lillehammer, and Halden explicitly consider the sustainability of the events conceptualised broadly. According to the definition given in this paper, this would require the municipalities to adopt “a holistic view of society” when regulating their events. In the Oslo case, the explicit consideration, however, is limited to the ecological dimension if one ignores the implicit social and economic sustainability ingrained in the city's general vision of being “green, warm and more creative” (Oslo Byråd, 2018, p. 1).

In Halden and Lillehammer, the policies combine their commitment to a broad conceptualisation with a commitment to a narrow conceptualisation of the events. In Halden, the policy makers have achieved this reconciliation by setting certain demands concerning the events' social, economic, and environmental impacts as a baseline for supporting the events (Halden Kommune, 2019, p. 2). In other words, Halden, along with the Lillehammerregion, require that event organisers consider the triple bottom line when planning their events (Halden Kommune, 2019, p. 3; cf. Lillehammerregionen, 2019, p. 6). In Halden, the overall aim is even to be “nationally recognised for developing and staging sustainable events of a good quality” if also as part of the local business development (Halden Kommune, 2019, p. 2; Hovedutvalg for samfunnsutvikling og kultur, 2019, p. 1).

Just as it is the case for the policies without explicit references to sustainability, one will have to conduct further studies to see if and how Halden, Oslo, and the Lillehammer region realise their aims and manage to reconcile the broad and narrow sustainability conceptualisations.

In summary, the event policies, to some degree, all conceptualise sustainability narrowly, i.e., aiming at securing a continuous staging of events. Further, picking up the remark from the previous section that one runs the risk of neglecting the broad conceptualisation of sustainability in an event policy, this does seem to be the case as only a minority of the event policies included a broad conceptualisation of sustainability.

Including the proceedings in the analysis might partly explain this focus. In most of the hearings, the primary driver for making the policy turned out to be a requirement for a more efficient handling of events from branch representatives. In Bergen, the proceedings for instance show that event representatives asked for an increased focus on security and a strategic approach to event organisation (Bergen Kommune, 2017). In Frederikstad and Tromsø, it was also a request that an event policy should make it less complicated to host events (cf. Tromsø Kommune, 2017; Frederikstad Kommune, 2019). The inputs on sustainability across all the hearings from non-event-organisers were few and mainly came from other municipalities (in the hearing on the regional policy for Lillehammer) and a few private actors in the Oslo case (not considering the specific hearing on sustainable events initiated by the municipality) (Oslo Kommune, 2018; Lillehammer Kommune, 2019). There were only few participants from the national sport organisations across the cases, and only the handball federation and the snowboard federation participated in the hearing in Oslo focusing on practical issues (Oslo Kommune, 2018, pp. 3, 67).

Overall, the very sources of influences fit well with the proposed model for influence on the event policies presented earlier (cf., Figure 1). The analysis further shows that the horizontal influence seems to be mainly concerned with event-specific issues with less of a focus on sustainability in a broad sense and vice versa for the vertical influence. These conclusions are however drawn on a very limited amount of data.

Summing up, the analysis shows that all the master plans conceptualise sustainability broadly. Several of the master plans acknowledge the SDGs as guidelines for the local policy development and others indicate a will to adopt a holistic perspective making references to the triple bottom line approach.

Looking at the business policies and the event policies, several of the policies conceptualise sustainability in a narrow sense. In these policies, “sustainable” events refer most often to an aim of making events reoccur regularly.

## Discussion: The Sustainability Puzzle

In other words, sustainability with regard to events is an unfinished puzzle for policy makers and when scrutinising the event policies, it becomes clear that the pieces in the event policy puzzle rarely fit perfectly with the master plan. How can one explain this observation and what are the potential implications?

The simple explanation would be that some event policies are simply in need of an overhaul and, in time, the policies will be reconciled. However, such an explanation disregards the character of the problem that the master plan and an event policy (eventually) conceptualising sustainability broadly have to solve. The great variation in conceptualisations found in the paper could namely indicate that there is no obvious solution to the problem. In other words, the problem is wicked.

Regarding the problem for the policies to solve as wicked nuances the observed reoccurring misfit between master plans and event policies. Instead of simply saying that some event policies are in need of an update that would solve the problem, one could see that policies as attempts to improve the solution of an existing problem.

To be more specific, the overall great variation in conceptualisations between the policies (some event policies conceptualising “sustainability” broadly and others in a narrow sense) indicates that the Norwegian municipalities are trying to improve the coherency of their event puzzle. However, as they lay the pieces of the puzzle, the pieces change and, with them, the look of the to-be-completed puzzle requiring the pieces to be laid again. The result is a flux that helps explain why some policies remain vague in their demands. Broad demands after all accommodate new inputs (or new pieces in the puzzle) more easily.

Still, the vagueness does not prevent a policy from making sense. Policy makers do not need have a clear picture of the final look of the puzzle, but they should know when it is improving and when it is not. A “good” policy according to Winship's puzzle-approach is after all a coherent policy not necessarily the most efficient.

What then does it take to make a policy coherent? Basically, it requires a willingness from the stakeholders to take inspiration and develop their perspectives, i.e., enter negotiations. In the case of the present study, the attempts to be coherent have sometimes led to event policies with a narrow sustainability conceptualisation, sometimes not. In either case, the (limited) analysis of the policy proceedings supports the idea that event policies act “in conjunction with [other] public policies” (Smith, 2012, p. 14).

However, the demand for efficiency in handling for instance the climate change raises the question whether the problem of sustainability will remain a wicked problem. As the problem of the climate change becomes more expressed, concrete, and obvious, the pressure to take precautions or adopt concrete solutions increases and future sport event policy makers might find it relevant to draw on another policy-event-relation proposed by Smith (2012), namely, “polices on events,” i.e., policies aiming at regulating events as a supplement or replacement of the currently prevalent “policies and events”—model aimed at using events as leverage. The inspiration for such regulation could come from the international frameworks mentioned in the introduction and context section. These frameworks offer a common ground for municipalities and their event partners on which to align their puzzle pieces and become coherent. The SDGs are already part of the Norwegian puzzle whereas international standards for sustainable events, such as the ISO 20121 for sustainable events or ISO 22379 for citywide events (under development), are as of today are missing as pieces.

Also, one could decide to replace the event pieces in the puzzle with other pieces with images (aims) similar to those of international events but with fewer potential negative impacts (cf. Taks, 2016). However, the current high interest in events does not speak for this solution.

## Contribution To Research

This paper is one of the first to consider the local relations between sport events and sustainability from a policy perspective. Its most important contribution to the research is therefore highlighting new relevant areas for further studies. For one, the paper shows the relevance of adding a sustainability perspective to the current research in event policies. Therefore, it opens a wider field of research on the explicit relation between sport events and sustainability, which is likely to become more relevant as the SDGs gain momentum.

The paper however also has its limits which future studies on specific events and their relation to the local event policies could mitigate. It has, for instance, not been able to consider the effect of the current policies. Especially given that some of the event policies are dated, studies of the concrete policy practise would give a more precise picture of how local event stakeholders handle sustainability. More generally, studies of the actual practise would also show whether the municipalities have the will and the resources to implement the sustainability aims stated in their event policies when faced with partners with potentially diverging agendas.

Finally, the study is based on event policies, which are meant to regulate both sport events and other cultural events. Currently, most event research focus on sport events (Bocarro et al., 2017). However, research based on local policies shows an intriguing field in which it is possible to study both sport and other forms of events within the same framework. In the future, this could lead to greater exchange between the various fields of event studies.

## Conclusion

The present paper analysed how 13 Norwegian municipalities and two regions conceptualise sustainability in their sport event hosting policies and discussed the potential explanations for and implications of these conceptualisations. The paper thereby highlighted the complexities municipalities could face when being interested in both hosting more sport events and increasing the sustainability of the municipality.

The paper first showed that the municipalities varied in how they conceptualised sustainability in their master plans and specific event policies. On the one hand, all the municipalities conceptualised sustainability in a “broad” sense in their master plans. In other words, the municipalities all agreed that local policy development should take the impacts of the policies on the local society as a whole into consideration. On the other hand, the paper's analysis of the specific event policies showed that these policies did not reflect the conceptualisation set out in the master plans. On the contrary, the specific policies mostly conceptualised sustainability as what the paper defined as “narrowly.” Thus, instead of considering sustainable events as something relating to how the event would impact the whole of the local society, the event policies considered events as “sustainable” when they occur on a regular basis.

This variation indicates that the reconciliation of sport events with a broad conceptualisation has no obvious solution. In other words, the problem could be considered as “wicked,” implying that any solution to the problem can only be an improvement on the existing approach and not final. Future research and practitioners should thus look to improve the existing solutions. This could include studying cases of best practise, but more detailed knowledge on the negotiations would be just as relevant since the paper, drawing on the puzzle-theory (Winship, 2006), points out that what eventually is adopted as a policy most likely also has to with the policy's ability to appear coherent and not necessarily best practise.

## Data Availability Statement

The raw data supporting the conclusions of this article will be made available by the authors, without undue reservation.

## Author Contributions

The author confirms being the sole contributor of this work and has approved it for publication.

## Conflict of Interest

The author declares that the research was conducted in the absence of any commercial or financial relationships that could be construed as a potential conflict of interest.

## Publisher's Note

All claims expressed in this article are solely those of the authors and do not necessarily represent those of their affiliated organizations, or those of the publisher, the editors and the reviewers. Any product that may be evaluated in this article, or claim that may be made by its manufacturer, is not guaranteed or endorsed by the publisher.

## Footnotes

^1^The parentheses around sport are there to indicate that the policies do not limit themselves to sport events in their wording. Still this is mainly a study on sport events as the municipalities' focus in praxis is on sport events (Jensen, 2020), which is also the most developed field of event research (Bocarro et al., 2017). Still, there are also research pointing out the similarities between sport events and events as such (Bowdin, 2012; Getz, 2012) and it will be discussed, in the conclusion, if and how the findings could contribute to the research on events more broadly.

^2^i.e., event strategy in Norwegian.

^3^Bergen, Kristiansand, Oslo, Stavanger, Tromsø og Trondheim (Innovasjon Norge, 2011, p. 5).

^4^Tinn Municipality figured in the list of results from the search among ^*^.kommune.no, however the municipality has 2019 only allocated money for the development of an event policy and is thus not included in the analysis (Tinn Kommune, 2019). Ørland Municipality figured too but is not included in the analysis as the relevant document only proposed the making of an event policy (Nye Ørland Kommune, 2019).

^5^Covering the Etne, Haugesund, Karmøy, Sveio, Tysvær and Vindafjord Municipalities.

^6^Covering the Gausdal, Lillehammer and Øyer Municipalities.

^7^To be precise, the social part (“samfunnsdel”) of the master plans (“kommuneplaner”) have been collected for all the municipalities.

## References

[B1] Amundsen H. Hovelsrud G. K. Aall C. Karlsson M. Westskog H. (2018). Local governments as drivers for societal transformation: towards the 1.5°C ambition. Curr. Opin. Environ. Sustain. 31, 23–29. 10.1016/j.cosust.2017.12.004

[B2] Andersson T. D. Getz D. Gration D. Raciti M. M. (2017). Event portfolios: asset value, risk and returns. Int. J. Event Festiv. Manage. 8, 226–243. 10.1108/IJEFM-01-2017-0008

[B3] Antchak V.. (2017). Portfolio of major events in Auckland: characteristics, perspectives and issues. J. Policy Res. Tour. Leisure Events 9, 280–297. 10.1080/19407963.2017.1312421

[B4] Antchak V. Ziakas V. Getz D. (2019). event portfolio management: theory and methods for event management and tourism. Goodfellow 8:4198. 10.23912/978-1-911396-91-8-4198

[B5] Baade R. A. Matheson V. A. (2016). Going for the gold: the economics of the olympics. J. Econ. Perspect. 30, 201–218. 10.1257/jep.30.2.201

[B6] Bell S. Morse S. (2018). A basis for systemic sustainability measurement, in Routledge Handbook of the History of Sustainability, ed J. L. Caradonna (London: Routledge), 187–218.

[B7] Bergen Kommune (2015). Bergen 2030. Berge: Kommuneplanens samfunnsdel.

[B8] Bergen Kommune (2017). Plan for store arrangement 2019–2030 [Saksframlegg 2017/11277-3]. Bergen: Bergen Kommune.

[B9] Bergen Kommune (2018). Plan for store arrangement 2019-2030. Bergen: Bergen Kommune.

[B10] Bocarro J. Byers T. Carter L. (2017). Legacy of sporting and non-sporting mega event research, in Legacies and Mega Events: Fact or Fairy Tales?, eds I. Brittain, J. Bocarro, T. Byers, and K. Swart (London: Routledge), 1–74.

[B11] Borowy I.. (2018). Sustainable development and the UN, in Routledge Handbook of the History Of Sustainability, ed J. L. Caradonna (London: Routledge), 151–163

[B12] Bowdin G. A. J.. (2012). Events Management (3rd ed.). London: Routledge Ltd - MUA.

[B13] Bowen G. A.. (2009). Document analysis as a qualitative research method. Qual. Res. J. 9:27. 10.3316/QRJ090202729062514

[B14] Buscarini C. Cerroni F. Gabrielli S. F. (2021). Structuring sustainable sport events and achieving desirable impact on urban development, in Sport Governance and Operations, London: Routledge.

[B15] Caradonna J. L.. (2018). Routledge Handbook of the History of Sustainability. London: Routledge.

[B16] Chalip L.. (2014). From legacy to leverage. In J. Grix (Ed.), *Leveraging Legacies From Sports Mega-events: Concepts and Cases* (pp. 2–12). New York, NY: Palgrave MacMillan.

[B17] Chappelet J.-L. Lee K. H. (2016). the emerging concept of sport-event-hosting strategy: definition and comparison. J. Global Sport Manage. 1, 34–48. 10.1080/24704067.2016.1177354

[B18] Clark R. Misener L. (2015). Understanding urban development through a sport events portfolio: a case study of London, Ontario. J. Sport. Manage. 29, 11–26. 10.1123/JSM.2013-0259

[B19] Collins A. Jones C. Munday M. (2009). Assessing the environmental impacts of mega sporting events: two options? Tour. Manage. 30, 828–837. 10.1016/j.tourman.2008.12.006

[B20] Dale G.. (2018). Sustaining what? scarcity, growth, and the natural order in the discourse on sustainability, 1650–1900. in Routledge Handbook of the History of Sustainability, ed J. L. Caradonna, (London: Routledge), 71–95 .

[B21] Deloitte (2018). Fra globale mål til lokal handling. Deloitte: Nordisk rapport 2018 =, 20.

[B22] Det Norske Akademis ordbok. (2019). Bærekraftig, in Det Norske Akademis ordbok. Det Norske Akademi. Available online at: https://naob.no/ordbok/b%C3%A6rekraftig?elementId=58281416 (accessed September 8, 2020).

[B23] Dunn W. N.. (2018). Public Policy Analysis: An Integrated Approach (Sixth Edition). Routledge: Taylor and Francis Group.

[B24] Elkington J.. (2004). Enter the triple bottom line, in The *Triple Bottom Line: Does It All Add Up*, eds A. Henriques, J. Richardson, and J. Richardson. London: Routledge.

[B25] Escher I.. (2020). Sustainable development in sport as a research field: a bibliometric analysis. J. Physic. Educ. Sport 20, 2803–2812. 10.7752/jpes.2020.s538133809896

[B26] Fossheim S. M. Nazareno M. S. (2020). Wicked problems og framtidige planutfordringer: Bærekraftig stedsutvikling i rammen av Regional plan for areal og transport i Oslo og Akershus [Norwegian University of Life Sciences, Ås]. Available online at: http://hdl.handle.net/11250/2679778 (accessed October 03, 2021).

[B27] Frederikstad Kommune (2018). Frederikstad mot 2030. Kommune: Kommuneplanens samfunnsdel.

[B28] Frederikstad Kommune (2019). Forslag arrangementsstrategi. Kommune: Fredrikstad kommune. Frederikstad Kommune.

[B29] Geeraert A.. (2018). N*ational sports governance observer. Final report. Play the Game: Danish Institute for Sports Studies*. Available online at: http://playthegame.org/knowledge-bank/downloads/national-sports-governance-observer-final-report/4c9cd824-ff3c-420e-bc9f-a996016f7d69 (accessed November 30, 2018)

[B30] Getz D.. (2012). Event Studies: Theory, research and Policy for Planned Events (2nd ed.). London: Routledge.

[B31] Gibson H. J. Kaplanidou K. Kang S. J. (2012). Small-scale event sport tourism: a case study in sustainable tourism. Sport Manage. Rev. 15, 160–170. 10.1016/j.smr.2011.08.013

[B32] Goldblatt D.. (2020). Playing Against the Clock: Global Sport, the Climate Emergency and the Case for Rapid Change. Glasgow: Rapid Transition Alliance.

[B33] Halden Kommune (2018). Kommuneplanens samfunnsdel. Kommune: Halden Kommune.

[B34] Halden Kommune (2019). Strategi for arrangementsbyen Halden. Kommune: Halden Kommune.

[B35] Hansen T.. (2020). Kommunalt selvstyre, in Store Norske Leksikon. Available online at: http://snl.no/kommunalt_selvstyre (accessed January 29, 2021).

[B36] Hanssen G. S.. (2021). FNs bærekraftmål, styring og samstyring. Verksted for regional utvikling. Available online at: https://www.regjeringen.no/contentassets/2a885633c4d1416282d5b17662d99006/forskernotat-fns-barekraftmal-styring-og-samstyring.pdf (accessed January 24, 2021).

[B37] Haugaland Vekst I. K. S.. (2017). Utviklingsplan for Haugesundregionen 2017-2020. Haugesund: Haugaland Vekst IKS.

[B38] Haugaland Vekst I. K. S.. (2019). Regional reiselivsstrategifor Haugalandet. 2019-2025. Haugesund: Haugaland Vekst IKS.

[B39] Higham J.. (2018). Sport Tourism Development. Bristol: Channel View Publications.

[B40] Hovedutvalg for samfunnsutvikling og kultur (2019). Arrangementsstrategi for Halden kommune (2018/5410-19). Kommune: Halden Kommune

[B41] Innovasjon Norge (2011). Hvitebok for arransjementsturisme. Innovasjon Norge. Available online at: http://www.innovasjonnorge.no/globalassets/old/pagefiles/27145/hvitebok-for-arrangementsturisme_web.pdf (accessed September 22, 2017).

[B42] Innovasjon Norge (2019). Norge som bærekraftig og innovativt arrangørland. Nasjonal Arrangementsstrategi 2020–2030.

[B43] IOC (2014). Olympic Agenda 2020. 20+*20 Recommendations*. Paris: Internatinal Olympic Committee. Available online at: https://stillmed.olympic.org/media/Document%20Library/OlympicOrg/Documents/Olympic-Agenda-2020/Olympic-Agenda-2020-20-20-Recommendations.pdf

[B44] ISO (2012). 20121: 2012 Event sustainability management systems - Requirements with guidance for use. ISO.

[B45] ISO/TC 292. (2019). ISO 22379 Security and resilience—Guidelines for hosting and organizing large citywide events. ISO/TC 292 Online. Available online at: http://www.isotc292online.org/projects/iso-22379/ (accessed November 8, 2019).

[B46] Jensen C. T.. (2020). Exploiting the spectacular. A study of Danish and Norwegian event stakeholders' interest in international sport events 2010-2020. [Doctoral thesis]. University of South-Eastern Norway.

[B47] Jiménez-García M. Ruiz-Chico J. Peña-Sánchez A. R. López-Sánchez J. A. (2020). A Bibliometric Analysis of Sports Tourism and Sustainability (2002–2019). Sustainability 12:2840. 10.3390/su12072840

[B48] Johnson R. B. Onwuegbuzie A. J. Turner L. A. (2007). Toward a definition of mixed methods research. J. Mix. Methods Res. 1, 112–133. 10.1177/1558689806298224

[B49] Karmøy Kommune (2020). Regionale reiselivsmidler (20/2515). Karmøy Kommune; Karmøy Kommune/*U*64.

[B50] Kim H.. (2020). Sustainability of the PyeongChang 2018 Winter Olympics [Doctoral, Manchester Metropolitan University]. Available online at: http://e-space.mmu.ac.uk/626132/

[B51] Koenigstorfer J. Bocarro J. N. Byers T. Edwards M. B. Jones G. J. Preuss H. (2019). Mapping research on legacy of mega sporting events: structural changes, consequences, and stakeholder evaluations in empirical studies. Leisure Stud. 38, 729–745. 10.1080/02614367.2019.1662830

[B52] Kommunal- og moderniseringsdepartementet (2019). Nasjonale forventninger til regional og kommunal planlegging 2019–2023. Kommunal: Kommunal-og moderniseringsdepartementet.

[B53] Kristiansand Kommune (2020). Kommuneplanens samfunnsdel. Kommune: Kristiansand Kommune.

[B54] Kulturdepartementet (2012). Den norske idrettsmodellen. Kommune: Kulturdepartementet.

[B55] Kulturdepartementet. (2019). Kunngjøring av prøveordning for arrangementsstøtte [Nyhet]. *Regjeringen.no*. Available online at: https://www.regjeringen.no/no/aktuelt/kunngjoring-av-proveordning-for-arrangementsstotte/id2673779/

[B56] Lechner E. Solberg H. A. (2021). The competition for government funding of major sports events: why do some applicants pass the needle's eye? Int. J. Sport Policy Politics 0, 1–15. 10.1080/19406940.2020.1859586

[B57] Lenskyj H. J.. (2004). The olympic industry and civil liberties: the threat to free speech and freedom of assembly. Sport Soc. 7, 370–384. 10.1080/1743043042000291695

[B58] Leopkey B. Ellis D. (2019). Sport event hosting capacity as event legacy: Canada and the hosting of FIFA events. Sport Bus. Manage. Int. J. 9, 45–62. 10.1108/SBM-09-2017-0047

[B59] Leopkey B. Mutter O. Parent M. M. (2010). Barriers and facilitators when hosting sporting events: exploring the Canadian and Swiss sport event hosting policies. Int. J. Sport Policy Politics 2, 113–134. 10.1080/19406940.2010.488058

[B60] Lillehammer Kommune (2019). Arrangementsstrategi for kommunene i Lillehammerregionen 2018-2022 [Saksframlegg 026 C03 18/9783-4]. Kommune: Lillehammer Kommune.

[B61] Lillehammer Kommunestyre. (2020). Protokoll-−*2020-01-23*. Lillehammer Kommune. Available online at: https://www.lillehammer.kommune.no/ato/esaoff/document/mteprotokoll-mte-i-kommunestyret-den-23-01-2020.1515813.05596ad2a2.pdf

[B62] Lillehammerregionen (2019). Arrangementsstrategi for kommunerne i Lillehammer-regionen 2019-2023. Kommune: Lillehammerregionen.

[B63] Lindsey I. Darby P. (2019). Sport and the sustainable development goals: where is the policy coherence? Int. Rev. Sociol. Sport 54, 793–812. 10.1177/1012690217752651

[B64] Mair J. Whitford M. (2013). An exploration of events research: Event topics, themes and emerging trends. Int. J. Event Festiv. Manage. 4, 6–30. 10.1108/17582951311307485

[B65] Mallen C. Stevens J. Adams L. J. (2011). A content analysis of environmental sustainability research in a sport-related journal sample. J. Sport. Manage. 25, 240–256. 10.1123/jsm.25.3.240

[B66] McCloy C.. (2009). Canada hosts the world: an examination of the first federal sport hosting policy. Int. J. Hist. Sport 26, 1155–1170. 10.1080/09523360902941761

[B67] McCullough B. P. Kellison T. B. (2018). Routledge Handbook of Sport and the Environment. London: Routledge.

[B68] McCullough B. P. Orr M. Kellison T. (2020). Sport Ecology: conceptualizing an emerging subdiscipline within sport management. J. Sport. Manage. 34, 509–520. 10.1123/jsm.2019-0294

[B69] Melo R. Van Rheenen D. Sobry C. (2021). Sport tourism events and local sustainable development: an overview, in Small Scale Sport Tourism Events and Local Sustainable Development: A Cross-National Comparative Perspective, eds R. Melo, C. Sobry, and D. Van Rheenen (New York, NY: Springer International Publishing), 1–42.

[B70] Nærings- og Fiskeridepartementet (2017). Opplev Norge—Unikt og eventyrligt. Stortinget. Available online at: http://www.innovasjonnorge.no/globalassets/reiseliv/rapporter-og-pulikbasjoner/in-reiselivsstrategi-2014-2020.pdf

[B71] NIF (2019). Idretten vil! Langtidsplan for norsk idrett 2019–2023. Oslo: Norges Idrettsforbund.

[B72] Norges Fotballforbund (2020). Strategiplan 2020-2023. Oslo: Norges Fotballforbund.

[B73] Norges Golfforbund (2020). Virksomhetsplan 2020-2023. Norges Golfforbund. Available online at: https://www.golfforbundet.no/assets/ngf/files/topp_meny/om_ngf/virksomhetsplan-2020-2023.pdf

[B74] Norges Gym- og Turnforbund (2018). strategisk plan 2018-2022. Oslo: Norges Gym- og Turnforbund.

[B75] Norges Handballforbund (2019). Strategiplan. Available online at: https://www.handball.no/globalassets/nhf-sentralt/om-oss/visjon-og-verdier/strategiplan/2019-strategiplan-etter-handballtinget-2019.pdf

[B76] Norges Skiforbund (2016). Skipolitisk dokument 2016-2020. Norges Skiforbund. Available online at: https://www.skiforbundet.no/globalassets/02-felles---medier/02-dokumentarkiv-moter/01-skitinget/2016/skipolitisk-dokument-2016-2020.pdf

[B77] Nye Ørland Kommune (2019). Besluttet struktur for nye Ørland kommune. Oslo: Nye Ørland Kommune.

[B78] O'Brien D. Chalip L. (2008). Sport events and strategic leveraging: pushing towards the triple bottom line, in Tourism Management: Analysis, Behaviour and Strategy, eds A. G. Woodside and D. Martin (CABI), 318–338.

[B79] Oslo Byråd (2018). Arrangementsstrategi for Oslo Kommune. Oslo: Oslo Kommune. Available online at: https://tjenester.oslo.kommune.no/ekstern/einnsyn-fillager/filtjeneste/fil?virksomhet=976819837andfilnavn=byr%2F2018%2Fbr1%2F2018032992-1919106.pdf

[B80] Oslo Kommune (2018). Innspill fra arrangører [Vedlegg Byrådssak 164 av 28.06.2018]. Kommune: Oslo Kommune.

[B81] Oslo Kommune (2019). Vår by, vår framtid. Kommuneplan for Oslo 2018.

[B82] Pinson J.. (2016). From the Olympic dream to a down to earth approach: Lausanne's sports events hosting strategy. Sport Soc. 19, 828–839. 10.1080/17430437.2015.1108650

[B83] Preuss H.. (2007). The conceptualisation and measurement of mega sport event legacies. J. Sport Tourism 12, 207–228. 10.1080/14775080701736957

[B84] Purvis B. Mao Y. Robinson D. (2019). Three pillars of sustainability: in search of conceptual origins. Sustain. Sci. 14, 681–695. 10.1007/s11625-018-0627-5

[B85] Raworth K.. (2017). Doughnut Economics: Seven Ways to Think like a 21st-century Economist. Available online at: https://www.overdrive.com/search?q=5043A4A2-A5B5-43C9-BEDB-2871DCE93F40

[B86] Reed E. U.. (2018). Vi må spille lokalpolitikerne gode. CICERO. Available online at: https://cicero.oslo.no/no/posts/klima/vi-maa-spille-lokalpolitikerne-gode

[B87] Rittel H. W. J. Webber M. M. (1973). Dilemmas in a general theory of planning. Policy Sci. 4, 155–169. 10.1007/BF01405730

[B88] Sandnes Kommune (2016). Arrangementsstrategi for Sandnes Kommune 2016–2019. Kommune: Sandnes Kommune.

[B89] Sandnes Kommune (2020). Arrangementsstrategi og arrangementshåndbok. Available online at: https://www.sandnes.kommune.no/kultur-fritid/kultur/kulturarrangement/arangement-handbok/

[B90] Schmelzer M.. (2018). The growth paradigm. history, hegemony, and the contested making of economic growthmanship, in Routledge Handbook of the History of Sustainability, ed J. L. Caradonna (London: Routledge), 164–186.

[B91] Schnitzer M. Schlemmer P. Kristiansen E. (2017). Youth multi-sport events in Austria: tourism strategy or just a coincidence? J. Sport Tour. 21, 179–199. 10.1080/14775085.2017.1300102

[B92] Singsaas M.. (2020). Bærekraftig utvikling i små kommuner (TF-notat 13/2020). Telemarksforskning.

[B93] Skien Kommune (2015). Kommuneplan—Samfundsdel 2015-2020. Kommune: Skien Kommune.

[B94] Smith A.. (2009). Theorising the relationship between major sport events and social sustainability. J. Sport Tour. 14, 109–120. 10.1080/14775080902965033

[B95] Smith A.. (2012). Events and Urban Regeneration: The Strategic Use of Events to Revitalise Cities. London: Routledge.

[B96] Språkrådet Universitetet i Bergen. (2020). Berekraftig. In Nynorskordboka. Språkrådet og Universitetet i Bergen. Available online at: https://ordbok.uib.no/perl/ordbok.cgi?OPP=+berekraftigandant_bokmaal=5andant_nynorsk=5andnynorsk=+andordbok=nynorsk (accessed September 8, 2020).

[B97] Stopper M. Gnädinger J. Kempf H. (2011a). Standortstrategien mit und für Sportgroßveranstaltungen—Eine vergleichende Analyse von acht Ländern, in Europäische Sportmodelle: Gemeinsamkeiten und Differenzen in international vergleichender Perspektive, eds E. Emrich and W. Andreff (Cartersville, GA: Hofmann), 163–176.

[B98] Stopper M. Gnädinger J. Kempf H. (2011b). The Gain of Playing Host. A Comparison of National Policies for Hosting Major Sporting Events. Available online at: https://books.openedition.org/uop/713?lang=en

[B99] Sunnfjord kommune (2019). Interkommunal plan for klimaomstilling i kommunane Førde, Gaular, Jølster og Naustdal. Available online at: https://sunnfjord.kommune.no/_f/p1/i58d3e39e-c360-45ff-9f05-9a3c586e415e/plan-og-kunnskapsdel-interkommunal-plan-for-klimaomstilling-vedtatt-juni-2019.pdf

[B100] Sunnfjord Utvikling (2019). Strategisk Næringsplan for Sunnfjord. Perioden 2019–2022.

[B101] Tainter J. A.. (2018). "Understanding sustainability through history. resources and complexity,in *Routledge Handbook of the History of Sustainability*, ed J. L. Caradonna (London: Routledge), 40–56.

[B102] Taks M.. (2013). Social sustainability of non-mega sport events in a global world. Euro. J. Sport Soc. 10, 121–141. 10.1080/16138171.2013.11687915

[B103] Taks M.. (2016). The rise and fall of mega sport events: The future is non-mega sport events, in Ethics and Governance in Sport: The Future of Sport Imagined, eds Y. V. Auweele, E. Cook, and J. Parry (London: Routledge), 84–93.

[B104] Thomson A. Cuskelly G. Toohey K. Kennelly M. Burton P. Fredline L. (2019). Sport event legacy: a systematic quantitative review of literature. Sport Manage. Rev. 22, 295–321. 10.1016/j.smr.2018.06.01130556493

[B105] Tinn Kommune (2019). Budsjett 2019. Tiltakskatalog. Tinn Kommune.

[B106] Triantafyllidis S. Darvin L. (2021). Mass-participant sport events and sustainable development: Gender, social bonding, and connectedness to nature as predictors of socially and environmentally responsible behavior intentions. Sustain. Sci. 16, 239–253. 10.1007/s11625-020-00867-x

[B107] Tromsø Kommune (2017). Arrangementsstrategi for Tromsø Kommune. Kommune: Tromsø Kommune.

[B108] Trondheim Kommune (2009). Kommuneplanens samfunnsdel. Kommune: Trondheim Kommune.

[B109] Ulstein Kommune (2018). Strateginotat for ein reiselivsplan for Ulstein kommune. Kommune: Ulstein Kommune.

[B110] United Nations. (2020). Take Action for the Sustainable Development Goals. United Nations Sustainable Development. Retrieved 11 November 2020, from https://www.un.org/sustainabledevelopment/sustainable-development-goals/

[B111] Wang L. Westskog H. Selvig E. Amundsen H. Mygland R. (2018). Kortreist kvalitet. Hva betyr omstilling til et lavutslippssamfunn for kommunesektoren? insam as, Cicero, Civitas AS og KS. Available online at: https://www.kortreistkvalitet.no/wp-content/uploads/2019/01/kortreist-kvalitet-v6.pdf

[B112] Winship C.. (2006). Policy analysis as puzzle solving, in The Oxford Handbook of Public Policy, eds M. Moran, M. Rein, and R. E. Goodin (Oxford: Oxford University Press, 109–123.

[B113] World Commission on Environment Development (1987). Our Common Future. Oxford: Oxford University Press. Available online at: http://archive.org/details/ourcommonfuture00worl

[B114] Yin R. K.. (2018). Case study research, design and methods (6th ed.). New York, NY: SAGE.

[B115] Ziakas V.. (2010). Understanding an event portfolio: The uncovering of interrelationships, synergies, and leveraging opportunities. J. Policy Res. Tour. Leisure Events 2, 144–164. 10.1080/19407963.2010.482274

[B116] Ziakas V.. (2019). Embracing the event portfolio paradigm in academic discourse and scholarship. J. Policy Res. Tour. Leisure Events 11, 27–33. 10.1080/19407963.2018.1556861

[B117] Zimbalist A.. (2015). Circus Maximus: The Economic Gamble Behind Hosting the OLYMPICS and the World Cup. Washington, D.C: Brookings Institution Press.

[B118] Zimbalist A.. (2017). Rio 2016. Washington, DC: Brookings Institution Press.

